# Birthing partners - validation of a questionnaire to assess the well-being of support persons

**DOI:** 10.1186/s12884-025-07685-y

**Published:** 2025-05-10

**Authors:** Nadine Schmitt, Juliane Lamprecht, Sabine Striebich, Gabriele Meyer

**Affiliations:** Medical Faculty, Institute for Health and Nursing Science, Martin Luther University Halle-Wittenberg, Magdeburger Straße 8, 06112 Halle (Saale), Germany

**Keywords:** Support person, Childbirth, Well-being, Questionnaire, Validity

## Abstract

**Background:**

There is little research on the well-being of the persons giving support during childbirth and how they feel when doing this. The aim of this study is to validate a questionnaire that assesses the well-being of those support persons during childbirth. This publication focuses on the validation of the questionnaire; the development is described in more detail in a previous publication.

**Methods:**

After the questionnaire had been developed, it was sent online to a sample for validation. Subsequently, the internal consistency was determined to assess the reliability and the correlation with an external criterion in order to assess the criterion validity. Known-groups validation was used to assess the construct validity of the questionnaire. Linear regressions were carried out to analyse which variables influence well-being.

**Results:**

The results show good reliability and high criterion validity. The known-groups analyses identified group differences between the different birth modes regarding the different domains of well-being of the support persons. Factors influencing well-being are whether it is a first time or repeated support, whether the birthing woman is a primiparous or multiparous woman and whether the birth is vaginal or operative.

**Conclusion:**

Our results suggest that this questionnaire adequately captures important aspects of the well-being of support persons during childbirth. The questionnaire is designed for all birth experiences. In the case of negative experience it can be used to assess additional support and counselling and thus potentially promote the mental health of the support persons preventively.

**Supplementary Information:**

The online version contains supplementary material available at 10.1186/s12884-025-07685-y.

## Background

Childbirth experience is a critical but rarely investigated rite of passage that can be a turning point for psychosocial change [1]. It is an experience that many women have together with their partner, making this transition a unique event in a couple's relationship [2]. The psychological dimensions and effects of the birth experience in relation to the transition to parenthood have not yet been sufficiently researched [3]. It becomes obvious that birth also appears to be a decisive life event for men, which has an impact on their later short- and long-term psychological well-being [4].

Although interest in the fathers'contribution to birth has increased in recent years [4], fathers feel that women are seen as a priority and are therefore less likely to seek support [5]. During the Covid-19 pandemic, in some places, the expectant father was not considered an equal partner by the health system in terms of their involvement in the process of becoming parents [6–8]. So they were deprived of their ability to do what partners saw as their most important task, which was to support the other partner [6]. Partners play an active role in the birth of their child, their views influence the birth process and their own psychological well-being [4].

### Effects of childbirth experience on the partnership

The birth experience can play a crucial role in how new parents adjust to the challenges of the relationship and parenthood [1]. The majority of partners feel that their relationship with the woman improved as a result of their presence at birth [9]. A positive shared birth experience can strengthen the couple's relationship [10], as can reflecting on the birth experience together [3]. The birth experience affects relationship satisfaction and the relationship satisfaction can also act as a mediator between the birth experience and mental health [11].

### Effects of childbirth on mental health

A positive birth experience is associated with lower anxiety and depression symptoms in the birth partners [11], whereas a negative birth experience was associated with a higher rate of postnatal depressive symptoms and a lower level of postnatal sense of security [12]. The fathers'assessments of subjective birth stress significantly predicted depressive symptoms six months after the birth [13]. These ratings of subjective labour stress differed significantly among fathers whose partners had given birth by emergency caesarean section compared to fathers whose partners had given birth vaginally both with and without medication [13]. Similar to the mothers, the way fathers experience the birth can have an impact on their mental health. Hoffmann et al. confirm the finding that a low-intervention birth leads to a more positive birth experience for the father [4]. This in turn predicted a better psychological well-being of the man after the birth and a lower probability of developing symptoms of postpartum depression [4].

In addition to the depressive symptoms, fear of childbirth can arise in the partners. Fathers with childbirth fear perceive a birth as risky and dangerous for the health of the woman and the baby. They believe they cannot cope with the unknown process of childbirth, but the fear subsides after the birth of their child [14]. A meta-analysis found that the rate of paternal perinatal anxiety is higher than the WHO global prevalence rates for anxiety in men. This means that the transition to parenthood poses a greater risk of anxiety for men [15].

### Traumatic birth experience

In some cases, the negative birth experience even leads to symptoms of post-traumatic stress disorder (PTSD). In recent years, it has been increasingly found that the support partners can also experience the birth as traumatic, although less frequently than the mothers [16, 17]. In a study with 202 health-care practitioners, they stated that they had observed birth trauma in 34% of mothers and 25% of partners. The most frequently observed symptoms were re-experiencing in mothers (87%) and avoidance in partners (51%) [18]. Fathers have different risk factors for the development of PTSD than women. The factors found were lower job satisfaction, higher workload, lower education, less support for the woman giving birth, the fact that they were becoming fathers for the first time [17] and past traumatic events [19].

A traumatic birth experience can have a negative impact on the couple’s relationship. 29.8% of mothers and 26.9% of partners perceived the birth trauma as an impairment of the couple relationship [18]. Reasons for the negative effects on the relationship may be that couples experience the time after the traumatic birth differently, have different coping mechanisms and needs, avoid talking about the birth and thus deal with it in silence and separately [20]. For some couples, however, birth trauma can also lead to a strengthening of the relationship [21].

### Effects of the birth experience on the attachment to the child

Even if fathers begin to bond with their baby during pregnancy [22], fathers feel more distant from their baby after the birth than mothers [23]. As the fathers had less sensory experience with the foetus compared to the mother, the moment of birth is described as very important. They describe it as a kind of realisation moment that represents the transition to fatherhood [22]. The birth experience of the partners therefore has a direct impact on the attachment to the child. A negative birth experience is associated with poorer attachment to the child 14 months after birth. This association was twice as strong in fathers as in mothers and was mediated by symptoms of postpartum depression and anxiety in fathers [24]. Conversely, Hoffmann et al. found that a more positive birth experience led to a more secure attachment to the child six months after the birth [4].

As can be seen, the birth experience has a variety of effects on different areas of the partners'lives. However, systematic research into the needs of partners during birth and what they feel while supporting the birthing woman are hardly available. A scoping review on the experiences of partners [25] provided an overview of some *qualitative* studies of good quality. The included *quantitative* studies were of moderate to low quality. The underlying literature search of this scoping review yielded only one validated questionnaire on the experiences of English-speaking first-time fathers. The scoping review points out that systematic research should be carried out into what support people need to have as a positive birth experience [25].

The aim of this study is to validate a questionnaire that assesses the Well-being Of support PErsons durinG childbirth in German language (WOhlbefinden von BegleitPErsonen während der Geburt) (WOPEG). It is important to note that only two of the working groups cited in the background are dedicated to all partnership constellations [6, 11, 24]; the majority of studies only include fathers. In the questionnaire developed here, we chose inclusive language in order to address all support persons. The detailed description of the questionnaire development can be found elsewhere [26]. As the validation is the subject of this article, only a brief description of the development of the questionnaire is given below.

## Method

### Questionnaire development

Several steps were taken in the development of the questionnaire. First, we identified core domains during labour support by conducting a systematic literature search and qualitative interviews. The systematic literature research revealed four domains: intense feelings, role of support, staff support and becoming a father. The content of these four domains is described in detail in the aforementioned scoping review [25]. The interviews were conducted via video chat with four fathers and one mother aged between 30 and 37. The information provided by the interviewees confirmed the findings of the literature research.

In the second step, we created an item pool based on the literature research and the interviews and developed an initial draft questionnaire with 89 items. A 4-point Likert response scale was used (disagree (1) to strongly agree (4)). In order to avoid response tendencies, the ratings of negatively worded statements were reverse-scored. Higher scores therefore reflect greater well-being during birth. The questionnaire was made available online, contained instruction and information on the first page and was to be completed anonymously.

The third step was to evaluate the items (i.e. questions). Therefore we conducted in a third step a cognitive pretest using the think-aloud technique, in which the partners were asked to rate the items for relevance and comprehensibility (face validity). We then asked experts for their critical evaluation of the items as to whether they are correct in terms of content (content validity). Afterwards, we conducted a pilot test using a smaller version of the larger survey to come, in which the partners were asked to provide written comments on improving the questionnaire.

In a fourth step, we conducted an initial empirical test with subsequent item analysis and principal component analysis with subsequent revision of the items. For this purpose, the link to the online questionnaire was sent to 739 mothers with the request to send it to their birthing partners. 164 fully completed questionnaires were included in the analysis. As part of the item analysis and the principal component analysis, items that were too simple, too difficult, items with low discriminatory power and items with insufficient loadings on the factors were excluded. This led to a reduction to 31 selective items with high discriminatory power and high loadings in four domains, resp. five domains if the ‘interaction with medical staff’ is further subdivided into interaction with midwives and interaction with doctors. Additional file 1 contains an overview of the domains (questionnaire scales) and the related items in English translation. The first domain partner's ‘informedness’ consists of six Items. These items deal with the partner's subjective perception of knowing what is happening, what will happen and how the woman giving birth can be supported. The second domain addresses'interaction with medical staff'. Nine items ask about the interaction with the midwife and six items with the physicians. As there are sometimes considerable differences between midwives and medical staff, these two domains are sometimes treated as two separate domains in the following. The third domain asks about the feeling of'belonging'and consists of four items. These relate to the feeling of being part of the process, being able to fulfil one's own expectations and being able to support the woman giving birth. The fourth domain deals with the ‘feelings’ of the partners when supporting the birth. Here, six items inquire about anxiety, being overwhelmed, worry about the duration of the birth and the pain of the woman giving birth, as well as the need to suppress feelings that arise.

Steps two to fourth are described in the previously mentioned article on the development of this questionnaire. Item generation, item evaluation and item analysis are described in detail here [26].

### Study sample and data collection for validation

The final online questionnaire should now be validated with additional persons. For this purpose potential German-speaking participants were recruited via social media (WhatsApp, Instagram, Facebook), posters in places where parents can be found, maternity clinics, and private and professional contacts. The link to the questionnaire was sent out with the invitation to send it on to potential participants. Participants were briefly informed about the project on the first page and that their data will be processed anonymously and then statistically analysed. They were only asked to take part if they had attended a birth in a clinic in Germany in the last 5 years (inclusion criteria). We chose these criteria because support persons report different experiences with out of hospital birth [27] and to avoid distortions due to memory effects caused by too long a period of time. Informed consent was considered as expressed when participants start answering the questionnaire by selecting the “continue” button.

### Statistical analysis

SPSS software for Windows (Version 28.0, SPSS Inc., Chicago, IL, USA) was used to conduct the psychometric, regressive and descriptive analyses.

To determine the reliability of the questionnaire the internal consistency was used. The internal consistency of a questionnaire describes its homogeneity and is calculated for each scale and for the entire questionnaire. The Cronbach`s alpha coefficient ranges from 0 to 1, and values of 0.70 or more indicate satisfactory internal consistency [28].

To verify whether the questionnaire correlates with or predicts the underlying construct of well-being, the criterion validity was determined. Criterion validity is a method of test validation that examines the extent to which scores on a questionnaire correlate with external criteria [29]. To assess, participants were asked at the end of the questionnaire to rate their general well-being during labour on a 10-point Likert scale (point 1 means the lowest imaginable well-being and point 10 the highest). The mean value of all items was then correlated with this external criterion.

Known-groups validation was used to assess the construct validity of the questionnaire, i.e. the ability of the instrument to distinguish between subgroups known to differ in important socio-demographic or clinical variables [28]. The selection of subgroups was based on the systematic literature research, which revealed differences in well-being across the following subgroups. One-way analysis of variance (ANOVA) was used to compare scale scores between groups, and Bonferroni post hoc tests were used to determine between which groups significant differences were found. Group differences were evaluated by comparing mean scores on each of the four domains (scales) between persons whose child was born by vaginal birth, by planned caesarean section, by caesarean section that became necessary during labour, or by emergency caesarean section with full anaesthesia and vaginal-operative birth. For an ANOVA with five groups, a mean effect size of about f = 0.25, a significance level of 0.05 and a power of 0.8, the target sample size is 195 persons [30].

The group that attended a vaginal birth was also asked in which position the child was born and whether it was born in the birthing bed. The possible answers regarding the birthing woman’s position were lying on the back (birth in supine position), lying on the side, on all fours, standing, on the birthing stool, kneeling, squatting, sitting. The birth position variable was dichotomised subsequently: Birth in supine position versus birth in other position (lying on the side or upright). The question whether the child was born in bed was asked dichotomously with yes or no. To compare scale scores ANOVA was also used.

In order to test which variables could influence the well-being of the supporting persons during birth, linear regressions were carried out with the total mean value of all scales as the dependent variable. The socio-demographic and birth-related variables surveyed here were each tested as independent variables.

## Results

The characteristics of the study participants are displayed in Table 1. 175 people could be recruited for validation. The mean age is 34 (*SD* = 6) years and 87% of the support persons were male. One person selected diverse gender, the others assigned themselves as female. 80% of the participating support persons had a high educational level. 92% of them stated that the woman giving birth was their partner, the majority had a relationship of more than five years duration. The remaining women who gave birth included two daughters, three sisters, seven good friends, a daughter-in-law and two refugees who were supported by refugee helpers.

**Table 1:** Characteristics of the study participants (*n*=175)

Variables	*N* (%)
Sociodemographic variables	
Gender, male	154 (87)
High educational level	142 (80)
Living in partnership	159 (92)
> 5 partnership years	113 (70)
Birth-related variables	
First birth support	111 (62)
Primiparous	109 (61)
Vaginal birth	132 (70)
Vaginal-operative birth	18 (10)
Planned caesarean section	15 (8)
Necessary caesarean section	20 (10)
Emergency caesarean section	3 (2)
Birth in supine position	70 (55)
Birth in bed	99 (79)

High educational level = University degree or high school diploma

The majority of the persons supported a childbearing woman for the first time and the majority of the mothers were primiparous. 70% had a vaginal birth, 10% a vaginal-operative birth and 20% a caesarean section. Half of the births were in supine position and two-thirds in bed.

Cronbach`s alpha for the entire questionnaire was 0.93. The scales (domains) have Cronbach`s alpha values between 0.80 and 0.88, indicating good reliability. Table 2 shows the alpha values as well as the mean scores for each scale and for total scale. As can be seen, the scale with the highest approval is ‘belonging’ and the lowest approval was the scale ‘interaction with physicians’.

**Table 2:** Descriptions of domains (scales) and total scale

Scale	Number of items	*M (SD)*	*α*	*r*_*EX*_
Informedness	6	2.97 (0.68)	.84	
Interaction with midwife	9	3.09 (0.70)	.88	
Interaction with physicians	6	2.65 (0.77)	.82	
Belonging	4	3.30 (0.65)	.82	
Feelings	6	2.88 (0.80)	.85	
Total scale	31	3.00 (0.61)	.94	0.72***

Range: 1-4, were 1 was worst and 4 best alternative, α Cronbach's alpha, r_EX_ Correlation with external criterion

***p < 0.001

The external criterion - the requested overall well-being during birth support - and the mean value of the items correlate high (*r* = 0.72, *p* > 0.01, *n* = 128). This value indicates high criterion validity.

**Fig. 1:**
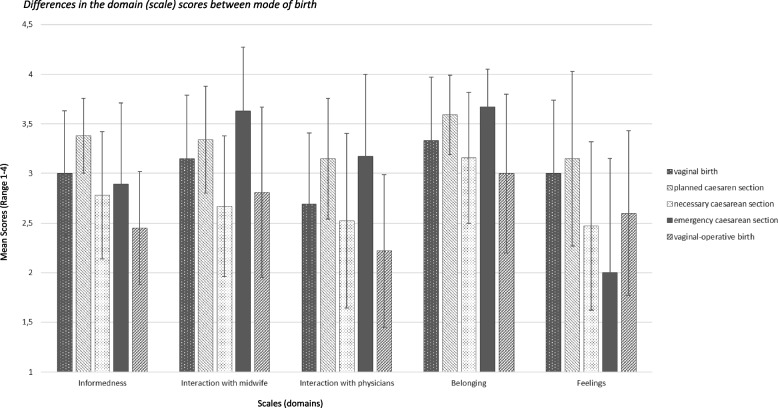
Differences in the domain (scale) scores between mode of birth

The results of the ANOVA indicate that the mean value of the scale scores differs between the different modes of birth (Fig. 1). The ‘informedness’ shows statistically significant differences for the various modes of birth, *F*(4,175) = 4.55, *p* < 0.01 as well as the ‘interaction with the midwife’, *F*(4,174) = 3.88, *p* < 0.01, the ‘interaction with the physicians’, *F*(4,168) = 3.34, *p* < 0.05 and the ‘feelings’, *F*(4,175) = 3.83, *p* < 0.01. There is no statistically significant difference in ‘belonging’ for the different modes of birth, *F*(4,174) = 2.05, *p* = 0.089. The effect sizes according to Cohen's f ranged were between 0.22 and 0.32 and are in the medium range [30]. Bonferroni post-hoc tests show a significant difference in ‘informedness’ between vaginal-operative births and vaginal births (−0.52, 95%-CI[−0.97, −0.07]) and planned caesarean sections (−0.89, 95%-CI[−1.55, −0.24]). Regarding the ‘interaction with the midwife’ Bonferroni post-hoc tests show significant differences in vaginal births and necessary caesarean Sects. (0.48, 95%-CI[−0.02, −0.93]). Regarding the ‘interaction with physicians’ Bonferroni post-hoc tests show significant differences in vaginal-operative births and planned caesarean sections (−0.93, 95%-CI[−1.72, −0.14]). Those who supported a planned caesarean section felt best informed and also indicated the most positive feelings. In the case of an emergency caesarean section, the support persons rated the interaction with both the midwife and the physicians as the most positive and their sense of belonging as the highest. But at the same time, these persons had the highest negative feelings about the situation. Those supporting a vaginal-operative birth rated the informedness, the interaction with the physicians and the feeling of belonging as being the lowest.

**Fig. 2:**
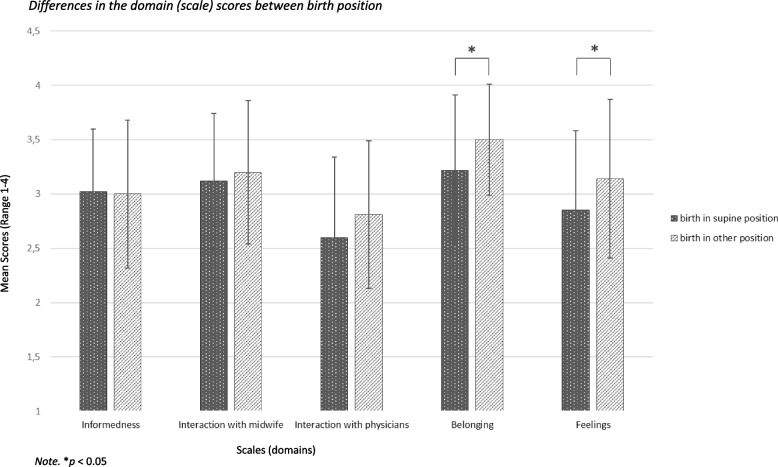
Differences in the domain (scale) scores between birth position

**Fig. 3:**
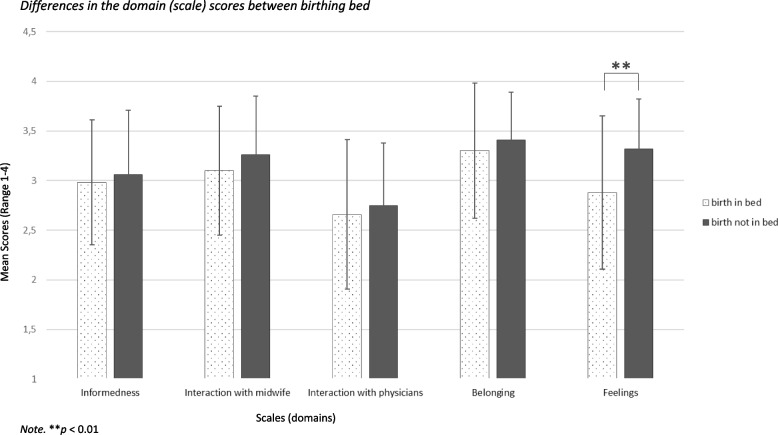
Differences in the domain (scale) scores between birthing bed

Figures 2 and 3 show the results of the ANOVA regarding the differences in the domain scores between birth position and birthing bed. There is no statistically significant difference in ‘informedness’ for births in supine or births in another position, *F*(1,125) = 0.03, *p* = 0.859 as well as births in bed or not in bed, *F*(1,124) = 0.20, *p* = 0.653. There is also no statistically significant difference in the ‘interaction with the midwife’ for births in supine or other positions, *F*(1,124) = 0.44, *p* = 0.508 and birth in bed or not, *F*(1,123) = 1.33, *p* = 0.252. Regarding the ‘interaction with physicians’ there is also no statistically significant difference for births in a supine or in another position, *F*(1,118) = 2.60, *p* = 0.110 or if births were in bed or not, *F*(1,117) = 0.31, *p* = 0.578. The sense of ‘belonging’ differed significantly between births in a supine position and other positions, *F*(1,124) = 6.47, *p* < 0.05 but not between births in bed or not in bed, *F*(1,123) = 0.68, *p* = 0.412. There is also a difference in ‘feelings’ between births in a supine position or other positions, *F*(1,125) = 4.97, *p* < 0.05 and also when births were in bed or not in bed, *F*(1,124) = 8.18, *p* < 0.01. The effect sizes are *f* = 0.23 for ‘belonging’ and *f* = 0.20 for ‘feelings’ and are in the medium range.

The result of the regression analyses (Table 3) is that the variables of first time or repeated birth support, primiparous or multiparous and vaginal birth or operative birth (vaginal-operative/sectio) have a significant influence on the well-being of the support persons. The birth position and the birth bed variable had no significant influence on well-being. Similarly, no socio-demographic variable had a significant influence on the well-being of the support persons. However, the effect sizes are negligible with values below 0.1, according to Cohen [31].

**Table 3:** Regression coefficients for predicting well-being

Variables	*B*	*95% CI*	*β*	*t*	*p*
Sociodemographic variables					
Age	.01	[-.01,-.03]	.14	1.80	.073
Gender	-.16	[-.41,.08]	-.10	-1.33	.407
Educational level	-.05	[-.16,.06]	-.07	-.91	.364
In partnership	-.20	[-.56,.16]	-.08	-1.09	.276
> 5 relationship years	.05	[-.14,.27]	.04	.52	.603
Birth-related variables					
First birth support	.20	[.04,.37]	.18	2.42	.038*
Primiparous	.20	[.04,.37]	.18	2.41	.017*
Vaginal birth	-.20	[-.38,.02]	-.16	-2.21	.029*
Birth in supine position	.15	[-.03,.32]	.15	1.64	.103
Birth in bed	.17	[-.04,.39]	.14	1.58	.116

*p < 0.05

## Discussion

This study reports on the validation of an instrument designed to assess the well-being of support persons during childbirth. Currently, the only available validated instrument assesses fathers` experiences of first childbirth [32]. It focuses on fathers rather than on support persons in general and only represents the perspective of the first experience. The aim of this study was to develop a questionnaire that includes the experiences of all support persons. Same-sex partners also want to be actively involved in the birth [33], but are confronted with barriers [34]. Their sexual orientation is sometimes prioritised by staff and they are not recognised in the language and procedures used [34].

The questionnaire was developed in several stages and validated on 175 support persons. The four domains and the questionnaire as a whole have a high internal consistency and therefore a high level of reliability. The instrument is therefore suitable for recording an overall value for well-being during the support given to a birthing woman, as well as reliably recording the constructs of ‘informedness’, ‘interaction with staff’, ‘belonging’ and ‘feelings’ at scale level. All in all, the mean values indicate high well-being for the population, considering the range from 1 to 4 for the overall score. There are differences in the mean values between the individual scales. The finding that the ‚interaction with physicians ‘ shows the least agreement is consistent with the findings from the interviews and the pretests. The first version of the questionnaire contained questions on interaction with medical staff that were not separated in professional groups. Both in the interviews and in the pretests, we received feedback that satisfaction with the care provided by the physicians and the midwives varied greatly. In German clinics, only the midwife is usually present during a physiological birth. A doctor is only called in if there are complications or at the end of the birth. The different approval ratings confirm that it is important to differentiate between professions in terms of well-being.

The criterion validity is high and indicates that the instrument can successfully measure the well-being of support persons. It can therefore be used as an initial screening instrument to detect potentially negative experiences. The days immediately after birth represent a window of opportunity for an early intervention for new fathers at risk of postpartum depression [13]. The first step in preventing mental illness is recognizing possible negative or even traumatic experiences. The developed instrument can serve as an initial screening tool to recognize these negative experiences and the related negative feelings. There is evidence that fathers of multiple births receive less professional and social support, whereas at the same time there is no difference in postnatal depression in fathers depending on parity [35]. The impact of the birth experience on mental health appears to be independent of whether it is a first time or repeated experience. For this reason, it is important that the instrument validated here also addresses people who experience a birth not for the first time.

Analyses of known groups showed that the instrument can distinguish between subgroups of support persons who are known to differ on important clinical variables, particularly birth mode or birth position. The finding that the support persons rated ‘interaction with staff’ and the sense of ‘belonging’ highest during an emergency caesarean section is consistent with the findings of [32]. This can presumably be attributed to the fact that an emergency caesarean section requires quick action and interaction between different professional groups. As expected, ‘feelings’ were rated lowest during an emergency caesarean section, meaning that staff take time to explain things to the support person and see the need for a flow of information. Nevertheless, there are also findings in the literature in which the emergency caesarean section is the reason for a poor birth experience [36]. Here, how this experience is evaluated seems to depend primarily on the explanations given by the staff, because when complications occur, support persons primarily need explanation, acknowledgement and reassurance [37].

Schytt and Bergström found that older men (over 34 years old) experience the pregnancy and birth more negatively than younger fathers [38]. Premberg et al. also showed that younger men received more emotional support and acceptance on the part of the staff [32]. We cannot confirm a difference in age. We also found no difference in terms of the gender, educational level or relationship status of the support person. Factors that influenced well-being in the present study were whether the person was supporting a birth for the first time or repeatedly, whether it was the first child of the woman giving birth, and the mode of birth.

As support persons have other risk factors for PTSD, they require individualised prevention and treatment services [17]. Negative aspects of support for support persons with PTSD were identified as: lack of help from friends and family, unhelpful postnatal debriefing services, lack of personal awareness of birth trauma, lack of trauma validation by healthcare professionals, lack of awareness of emotional needs, and barriers to accessing mental health services [21]. Mothers and their partners should be given special attention to postpartum PTSD and postpartum psychological distress [19]. As the entire family system can be affected by direct or indirect PTSD, further studies on partners PTSD are explicitly indicated [16]. Webb et al. have adapted the City Birth Trauma Scale for use with support persons [39]. The questionnaire developed and validated here goes one step further and aims to give this important group of people, the support persons, the attention they deserve. Even though the questionnaire can provide indications of a potential traumatic experience, it is primarily used to find out what support persons generally need in order to have a good experience.

The questionnaire focuses exclusively on the birth experience. The effects of the birth experience on support persons during the time after the birth and on relationship satisfaction are not part of this questionnaire. This could be the subject of later studies in order to establish connections between the birth experience and the postnatal effects. It is also important to mention that the non-birthing parent is an important resource for the mother and child after the birth, especially for mothers with postnatal depression [40]. It should therefore be a matter of urgency to promote the well-being of this group of people in order to provide this important support.

Although the present questionnaire fills a gap and there is now an instrument for investigating the well-being of persons who support women during childbirth for German-speaking countries, this limits its international applicability. Subsequent studies can test the proposed translation of the items and validate them on an international sample. A strength of the study is the diversity of recruitment, as analog methods were used in addition to social media to facilitate access to participation. However, the average sample size and the high proportion of people with a higher level of education should be mentioned as limitations. Possible distortions due to the limited sample should be taken into account when interpreting the results. Nevertheless, the comprehensive development and validation of the instrument has led to results that indicate that the instrument adequately measures well-being and can differentiate well between different persons.

## Conclusion

Support persons have become an essential part of the labour room, as they are an important part of the birth experience for the women giving birth. However, these people are not only very important for the women giving birth; the support persons also want to actively support the birth of the child and be involved in the beginning of parenthood. For this reason, there is now a validated questionnaire that measures the well-being of support persons. Our results suggest that this questionnaire adequately captures important aspects of support persons' well-being during labour and birth. It can serve as a useful tool to assess experiences and needs and can be used to assess additional support and counselling in the case of negative birth experiences. It can therefore be an important screening tool to mitigate potential negative effects of the birth experience on mental health.

## Supplementary Information

Additional file 1. English translation of the questionnaire scales and items.

## Data Availability

The data sets used and analysed in the current study are available on request from the corresponding author.

## Abbreviation

PTSD: Post-traumatic stress disorder
