# Supplementary material for: Birthing partners - validation of a questionnaire to assess the well-being of support persons
Source: BMC Pregnancy Childbirth. 2025 May 10;25:561. doi: 10.1186/s12884-025-07685-y (PMC12065179; doi:10.1186/s12884-025-07685-y)
Supplement: Supplementary file 1 — Additional file 1. English translation of the questionnaire scales and items. [file 12884_2025_7685_MOESM1_ESM.pdf]

**Additional file 1.** English translation of the questionnaire scales and items.
**Informedness**
I usually had the feeling that I knew what was happening.
I usually knew what I could do to support the woman giving birth.
I felt prepared for what can happen/has happened during the birth.
I felt prepared for what might happen/what happened during the birth.
Most of the time I knew how the birth would proceed.
At least once I had the feeling that I had no control over the situation.
**Interaction with the midwife**
I wish the midwife had listened to me more.
I felt supported by the midwife.
The midwife gave me enough information about what she is doing and will do.
I would have liked the midwife to involve me more in decisions.
The midwife showed me how to support the woman giving birth.
There were situations in which I would have liked the midwife to give me more information.
I would have liked the midwife to be in the room more often.
I felt involved in decisions by the midwife.
The midwife offered me something to drink or eat or asked me what I needed.
**Interaction with physicians**
The medical staff has informed me sufficiently about what it does and will do.
I would have liked the medical staff to show me what I could do.
I would have liked the medical staff to be present in the room more often.
The medical staff gave the woman giving birth and me the opportunity to help shape the birth.
I felt the medical staff involved me in decisions.
Someone from the medical showed me how to support the woman giving birth.
**Belonging**
I felt like I didn't belong.
I have fulfilled my own expectations of myself as a support person.
I felt myself to be involved in the birth.
I was able to give good support to the woman giving birth well.
**Feelings**
I felt the need to suppress any feelings that arose.
I felt that I had to suppress any feelings that arose.
I was overwhelmed by the situation.
I was afraid.
I was worried about the duration of the birth.
I was worried about the pain of the women giving birth.
